# Ultrasound versus magnetic resonance imaging for calculating total kidney volume in patients with ADPKD: a real-world data analysis

**DOI:** 10.1186/s13089-025-00400-0

**Published:** 2025-02-11

**Authors:** Juan M. Fernandez, Carmen Rosa Hernández-Socorro, Lucas Omar Robador, Francisco Rodríguez-Esparragón, Daniela Medina-García, Juan Carlos Quevedo-Reina, Mercedes Lorenzo-Medina, Elena Oliva-Dámaso, Patricia Pérez-Borges, José C. Rodríguez-Perez

**Affiliations:** 1 Baxter Healthcare Medical Department, Madrid, Spain; 2 PhD Programme in Biomedical Research at the University of Las Palmas de Gran Canaria (ULPGC), Luis Suárez Suárez 81, 35018 Las Palmas de Gran Canaria, Spain; 3 Radiology Department, Hospital Universitario de Gran Canaria Dr. Negrín (HUGCDN), Las Palmas de Gran Canaria, Spain; 4 Universidad Fernando Pessoa Canarias, Las Palmas de Gran Canaria, Spain; 5 Research Unit of HUGCDN, Las Palmas de Gran Canaria, Spain; 6 Nephrology Department of HUGCDN, Las Palmas de Gran Canaria, Spain; 7 Universidad de Las Palmas de Gran Canaria (ULPGC), Las Palmas de Gran Canaria, Spain; 8 Clinical Analysis Department of HUGCDN, Las Palmas de Gran Canaria, Spain

**Keywords:** ADPKD, Kidney volume, Ultrasonography, Magnetic resonance imaging, Glomerular filtration rate, Disease progression

## Abstract

**Background and objectives:**

This study aimed to compare Total kidney volume (TKV) measurements using US-ellipsoid (US-EL) and MRI-ellipsoid (MRI-EL) in patients with autosomal-dominant-polycystic-kidney-disease (ADPKD). It also evaluated whether the agreement between right (RKV) and left (LKV) kidney volume measurements differed.

**Methods:**

Retrospective analysis of a prospective data-base that included consecutive patients diagnosed with ADPKD. Total kidney volumes by 3D-US-EL were compared with those by MRI-EL. Bland–Altman-plots, Passing–Bablok-regression, and the concordance-correlation-coefficient (CCC) were used to compare right (RKV), left (LKV), and TKV measurements.

**Results:**

Thirty-two ADPKD patients, 14(43.7%) women, were included. Mean measured (mGFR) and estimated (eGFR) glomerular-filtration-rate (GFR) were 86.5 ± 23.9 mL/min and 78.9 ± 23.6 mL/min, respectively. Compared with MRI-EL, TKV (Mean difference: − 85.9 ± 825.6 mL; 95%CI − 498.5 to 326.7 mL; p = 0.6787), RKV (Mean difference: − 58.5 ± 507.7 mL; 95%CI − 312.2 to 195.2 mL; p = 0.6466), and LKV (Mean difference: − 27.4 ± 413.5 mL; 95%CI − 234.1 to 179.2 mL; p = 0.7918) were lower with US-EL than with MRI-EL, although without significant differences. According to Passing and Bablok-regression analysis, the Spearman correlation-coefficient was 0.96 (95%CI 0.92 to 0.98); 0.91 (95%CI 0.82 to 0.96), and 0.94 (95%CI 0.87 to 0.97) in the RKV, LKV, and TKV, respectively; p < 0.0001 each, respectively. CCC of RKV, LKV, and TKV measurements were 0.95, 0.89, and 0.94, respectively. The mGFR and eGFR showed statistically significant negative correlations with TKV measured by both MRI-EL (p = 0.0281 and p = 0.0054, respectively) and US-EL (p = p = 0.0332 and p = 0.0040, respectively).

**Conclusions:**

This study found that ultrasound-based ellipsoid kidney volume measurements strongly correlated with MRI-based measurements, suggesting that ultrasound is a reliable, accessible alternative for assessing kidney volume, particularly when MRI is unavailable.

**Supplementary Information:**

The online version contains supplementary material available at 10.1186/s13089-025-00400-0.

## Introduction

Autosomal dominant polycystic kidney disease (ADPKD) is the most common inherited renal disorder worldwide, characterized by the progressive formation of numerous cysts that compress the renal parenchyma, ultimately leading to end-stage renal disease (ESRD) in adulthood [1–4]. The disease arises from mutations in the PKD1 or PKD2 genes, with PKD1 mutations being more common and associated with earlier onset and more severe clinical manifestations compared to PKD2 mutations [4–6]. This genetic variability can complicate diagnosis, especially in younger patients with milder symptoms [5, 6].

Traditional markers of kidney function, such as serum creatinine, estimated glomerular filtration rate (eGFR), and creatinine clearance, are not reliable for assessing disease severity or progression in ADPKD. These parameters typically remain within normal ranges until the disease reaches advanced stages [6–8]. In contrast, findings from the Consortium for Radiologic Imaging Study of Polycystic Kidney Disease (CRISP) highlight total kidney volume (TKV) as a key biomarker. TKV in ADPKD increases in a quasi-exponential manner throughout adulthood, with an average annual growth rate of 5%, although individual variability is substantial [9]. Recognizing its predictive value, both the U.S. Food and Drug Administration (FDA) and the European Medicines Agency (EMA) have endorsed TKV as a prognostic biomarker for identifying patients at high risk of progression, facilitating inclusion in clinical trials [7, 9, 10].

The gold standard for TKV measurement involves magnetic resonance imaging (MRI) or computed tomography (CT) with manual segmentation, a labor-intensive and resource-intensive process requiring radiological expertise [11–13]. In contrast, ultrasound (US) offers a more accessible and cost-effective alternative. Using the ellipsoid formula (US-EL), kidney volume can be approximated by measuring three orthogonal axes, though this method is considered less precise [14]. Additionally, the availability of three-dimensional (3D) ultrasound in many tertiary care centers provides a promising tool for volumetric assessments and has shown potential for TKV quantification in non-ADPKD populations [15, 16].

The current study aimed to compare the agreement of TKV measurements assessed by US-EL versus (vs) MRI-ellipsoid in patients with ADPKD. Additionally, we also compared the volume measurements of the right (RKV) and left (LKV) kidneys individually for evaluating whether the degree of agreement differed between both kidneys.

## Methods

### Study design

This study was a retrospective analysis of a prospective database involving consecutive patients diagnosed with ADPKD, who were followed by the out-patient clinical office at the third-level University Hospital of Gran Canaria Doctor Negrín (HUGCDN) (Las Palmas de Gran Canaria, Spain).

All participants provided informed consent in accordance with a predetermined study protocol that received approval from the Ethics Committee of HUGCDN (Protocol VO 05-2017; Review Board approval: 170071; May 2017). This research adhered to the principles established in the Good Clinical Practice/International Council for Harmonization Guidelines, the Declaration of Helsinki, and all pertinent country-specific regulations governing clinical research, emphasizing the highest level of individual protection.

To maintain anonymity, any potentially identifiable information was either encrypted or removed from the dataset.

### Study patients

This study included patients > 18 years of age who were diagnosed with ADPKD based on ultrasound-3D or MRI criteria [17, 18], clinically stable [19], without acute kidney injury, and had an eGFR, assessed with the Chronic Kidney Disease Epidemiology Collaboration (CKD-EPI) formula, > 60 mL/min. In addition, patients had to have not indicate active infectious diseases or cardiovascular events within the 3 months preceding study enrollment.

Patients with a history of iodine allergy, contraindications for undergoing MRI, active malignancies, uremia or impending dialysis, severe psychiatric disorders, or those who were pregnant or breastfeeding were excluded from the study.

### Glomerular filtration rate (GFR)

#### Measured GFR (mGFR)

On the day of the study visit (baseline), a 5 mL intravenous injection of iohexol solution (Omnipaque 300, GE Healthcare) was given over a 2-min period. Iohexol concentrations were measured using dried blood spot (DBS) samples, which were then sent to the University Hospital of Canarias, La Laguna (Tenerife, Spain) for analysis [20]. Plasma clearance of iohexol was calculated using the method outlined by Krutzé et al. [21].

#### Estimated GRF

Simultaneously to the clearance of iohexol, the CKD-EPI formula [22] was used to calculate eGFR.

### Ultrasound-3D kidney imaging

Ultrasound examinations were conducted individually for each kidney utilizing a Aplio 500 US device (Canon Medical Systems Corporation, Tokyo, Japan), with 3.5 MHz mechanical convex D transducer. If the borders of the kidney were not fully captured within the imaging display, the lengths were measured using a panoramic function, also known as extended field of view ultrasound.

TKV by ultrasound-ellipsoid was assessed using the ellipsoid formula:

$$Volume = \frac{\pi }{6}* \left( {Heigth*Width*Length} \right)$$

The transducer was positioned in a longitudinal orientation along the upper pole of the kidney and then moved in a linear fashion down to the lower pole; the software subsequently dynamically “stitches” the images acquired during the transducer’s movement. All scans were evaluated by the same radiologist (CRHS) who was blinded to the clinical information of the participants.

The ellipsoid volume calculation utilized sagittal length (mm), coronal length (mm), width (mm), and depth (mm) measurements obtained from the MRI, according to the following formula [23]:

$$Volume = \frac{\pi }{6}* \left( {Heigth*Width*Length} \right)$$

No contrast material was used in any of the patients.

All MRI were analyzed by the same radiologist (LOR) who was blinded to both the clinical information of the participants and the ultrasound data.

The ultrasound-3D and MRI examinations were performed independently, with a maximum time between them of 9 months.

### Statistical analysis

Statistical analysis was conducted using MedCalc® Statistical Software version 23.0.2 (MedCalc Software Ltd, Ostend, Belgium; https://www.medcalc.org; 2024).

The Shapiro–Wilk test was employed to evaluate the normality of quantitative variables.

Continuous variables were presented as means and standard deviations (SDs), while non-normally distributed variables were reported as medians and interquartile ranges. Categorical variables were expressed as percentages along with 95% confidence intervals (95% CIs). To compare RKV, LKV, and TKV measurements Bland–Altman plots, Passing–Bablok regression, and the concordance correlation coefficient were used.

From the Bland–Altman plots, biases were calculated as the mean percentage differences from zero; a bias > 0.05 indicated no difference in the mean value of two measurement methods.

Passing and Bablok regression analysis was employed to evaluate the concordance between the MRI-EL and US-EL imaging methods for measuring RKV, LKV, and TKV. This non-parametric statistical approach is particularly effective for assessing the agreement between two analytical methods, providing insight into any systematic differences or proportional biases that may exist. If 95% CI for slope includes value one, it can be concluded that there is no significant difference between obtained slope value and value one and there is no proportional difference between two methods [24, 25].

To evaluate agreement between US-EL and MRI-EL, we calculated Lin’s concordance correlation coefficient (CCC) for the individual and TKV volumes [26, 27]. CCC values range between 0 and 1 and can be interpreted as follows: < 0.9 indicates poor agreement, ≥ 0.90 to ≤ 0.95 reflects moderate agreement, values > 0.95 to ≤ 0.99 represent substantial agreement, and values > 0.99 indicate almost perfect agreement [27].

## Results

### Baseline demographic, clinical, and analytical characteristics

This study included 32 ADPKD patients, 14 (43.7%) women and 18 (56.2%) men, with a mean age of 42.0 ± 15.8 years. Mean measured glomerular filtration rate (mGFR), assessed by plasma clearance of iohexol, was 86.5 ± 23.9 mL/min; while estimated GFR (eGFR) assessed by CKD-EPI formula was 78.9 ± 23.6 mL/min. Mean body mass index (BMI) was 24.6 ± 3.7 kg/m^2^.

**Fig. 1:**
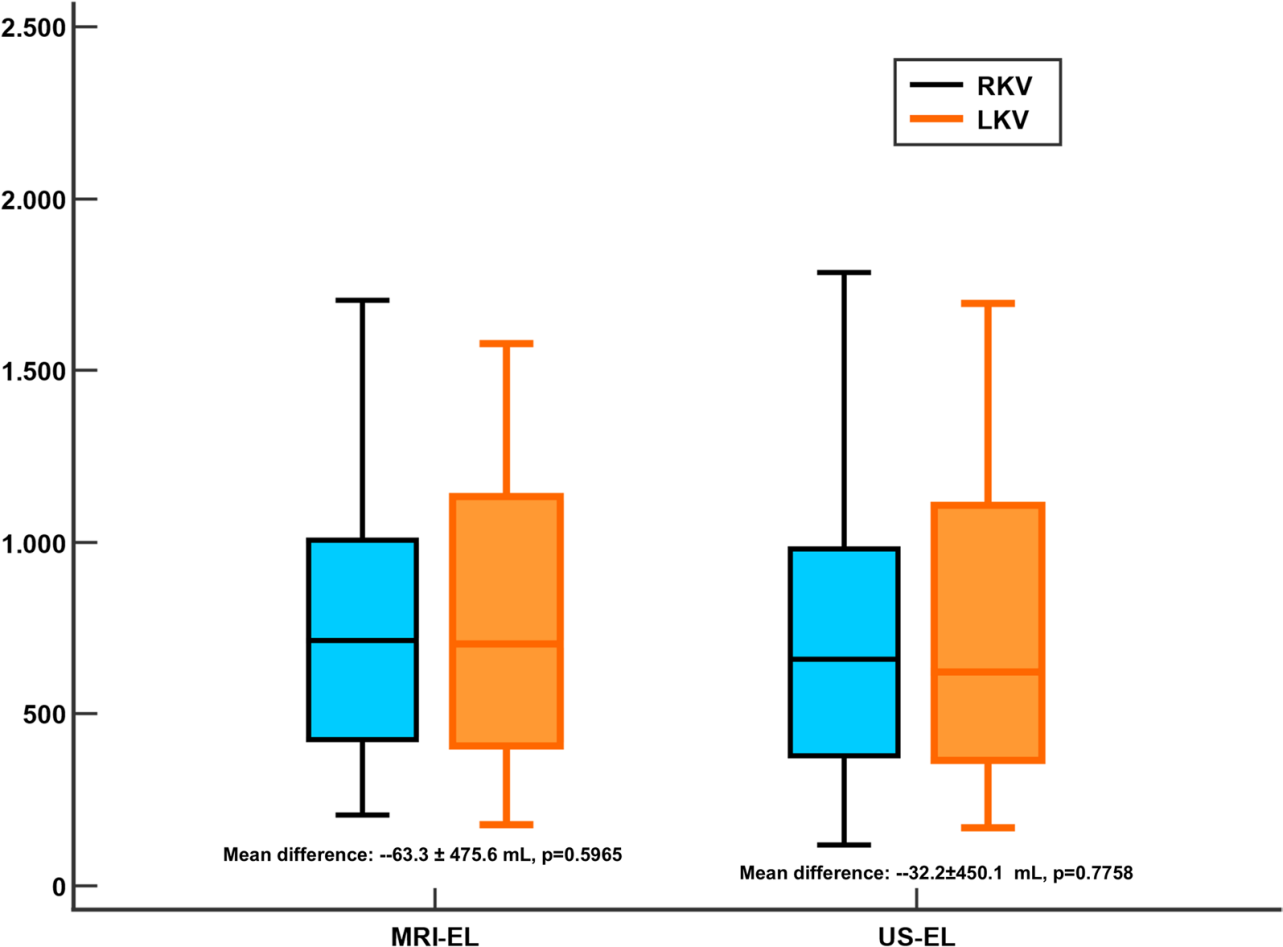
Box and whisker evaluating the difference between the right (RKV) (blue) and left (LKV) (Orange) kidney volumes assessed by magnetic resonance imaging ellipsoid (MRI-EL) and ultrasound ellipsoid (US-EL)

The US-El study found no differences between RKV (mean volume: 757.6 ± 485.5 mL; 95%CI 582.5 mL to 932.6 mL) and LKV (mean volume: 725.4 ± 411.7 mL; 95%CI 577.0 mL to 873.8 mL) measurements (mean difference: − 32.2 ± 450.1 mL; 95%CI − 257.1 mL to 192.7 mL; p = 0.7758). Similarly, MRI-EL demonstrated no significant differences in the measurements of RKV (mean volume: 816.1 ± 529.0 mL; 95% CI 625.3 mL to 1006.8 mL) and LKV (mean volume: 752.8 ± 415.3 mL; 95% CI 603.1 mL to 902.5 mL) (mean difference: − 63.3 ± 475.6 mL; 95%CI − 300.9 mL to 174.4 mL; p = 0.5965) (Fig. 1).

### Comparison of kidney volume measurements assessed by US-EL vs MRI-EL

**Fig. 2:**
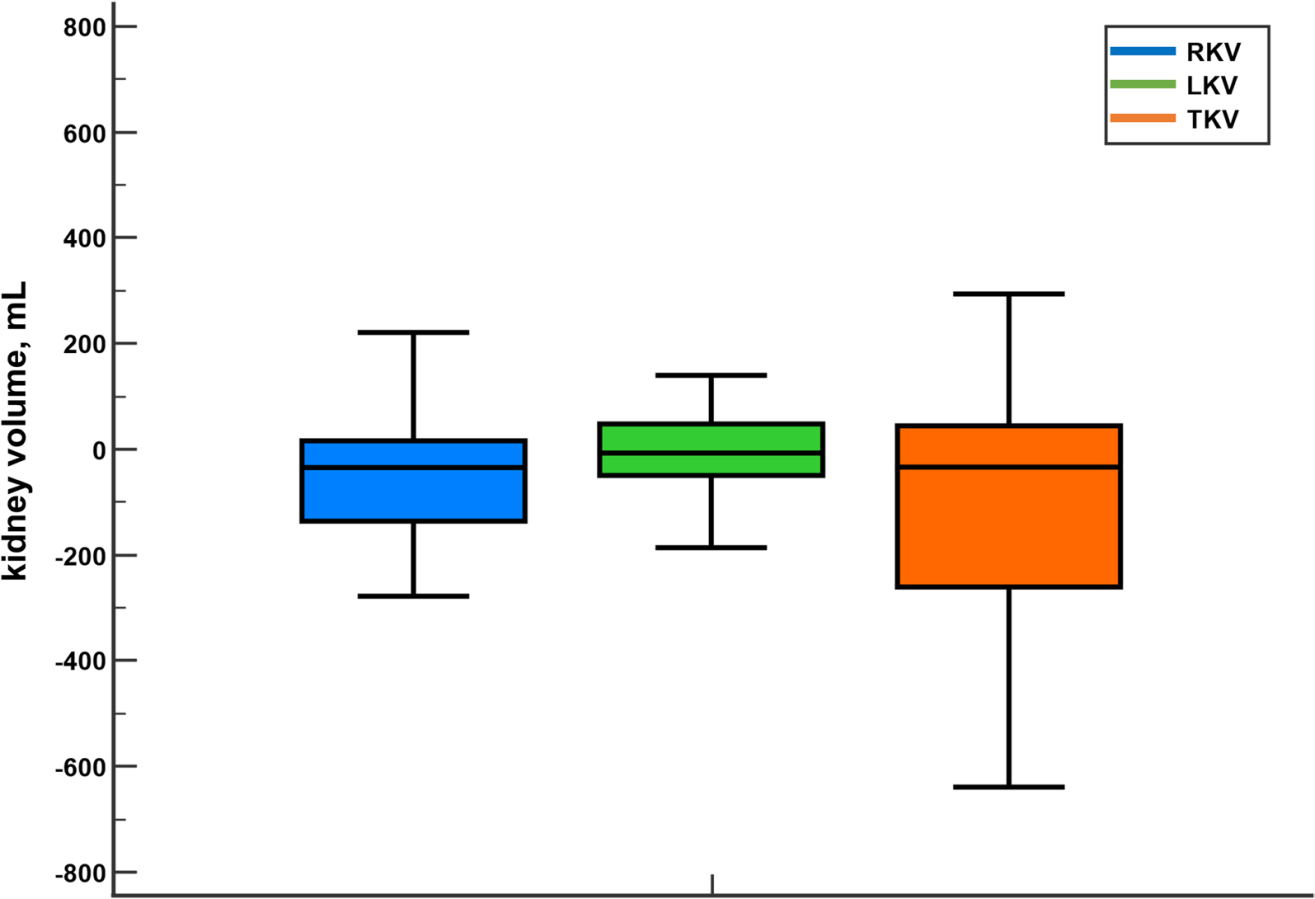
Box and whisker evaluating the difference between magnetic resonance imaging ellipsoid (MRI-EL) and ultrasound ellipsoid (US-EL) in the right (blue), left (green), and total (orange) kidney volume. Right kidney volume (RKV): Mean difference: − 58.5 ± 507.7 mL, p = 0.6466. Left kidney volume (LKV): Mean difference: − 27.4 ± 407.5 mL, p = 0.7918. Total kidney volume: Mean difference: − 85.9 ± 825.6 mL, p = 0.6787

Compared with MRI-EL (reference standard), kidney volumes measured with ultrasound-3D were smaller than those measured with MRI. These differences were more pronounced in the TKV (Mean difference: − 85.9 ± 825.6 mL; 95%CI − 498.5 to 326.7 mL; p = 0.6787), followed by RKV (Mean difference: − 58.5 ± 507.7 mL; 95%CI − 312.2 to 195.2 mL; p = 0.6466), and LKV (Mean difference: − 27.4 ± 413.5 mL; 95%CI − 234.1 to 179.2 mL; p = 0.7918); although in no case were these differences statistically significant (Fig. 2).

**Fig. 3:**
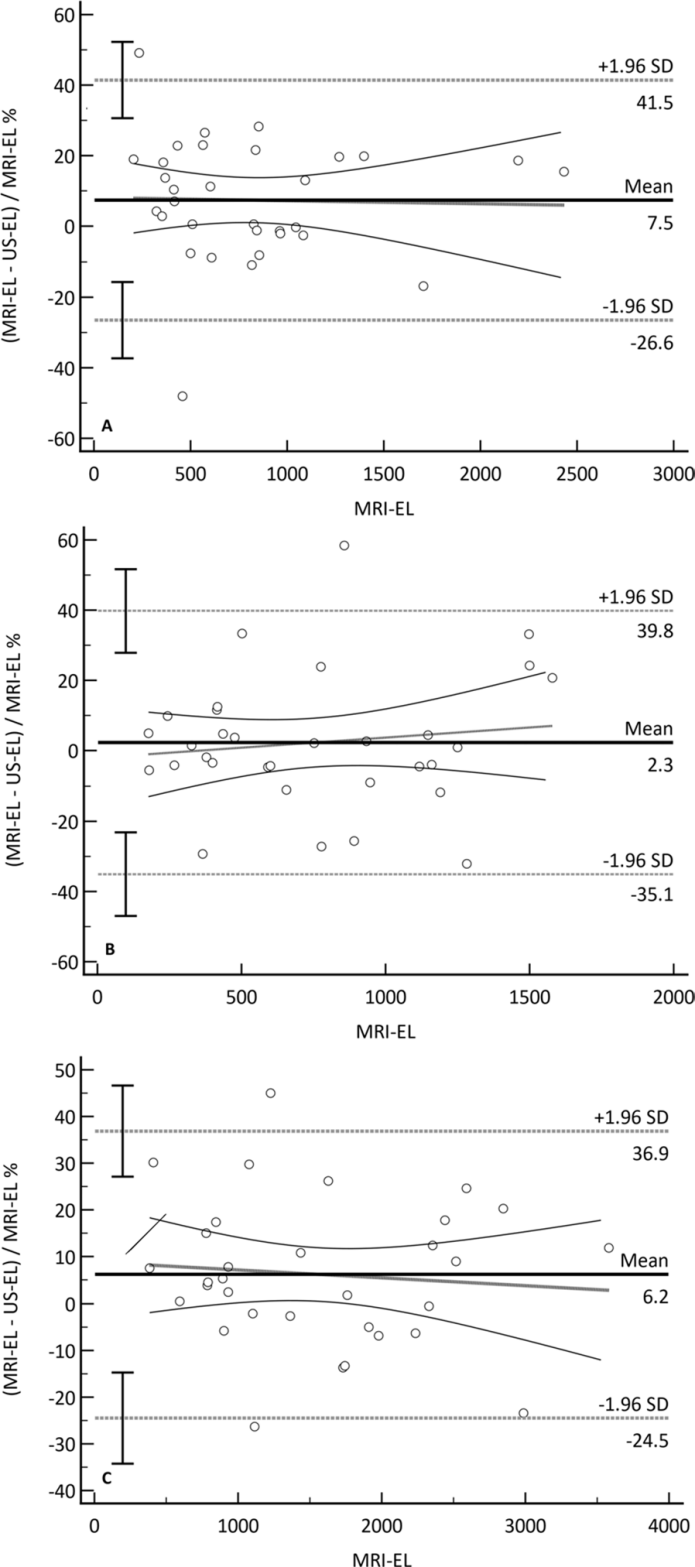
Bland–Altman plots showing within-patient differences of right kidney volume (**A**), left kidney volume (**B**), and total kidney volume (TKV) (**C**) measured by ultrasound ellipsoid (US-EL) in comparison with magnetic resonance imaging ellipsoid (MRI-EL) (reference standard). The solid black line represents the mean percentage difference (bias). The grey dotted lines are the 95% limits of agreement. The black dotted line is the slope of the bias with the 95%CI. Mean slope RKV: − 0.00; 95% CI − 0.01 to 0.01, p = 0.8874. Mean slope LKV: 0.01; 95% CI − 0.01 to 0.02, p = 0.4885. Mean slope TKV: − 0.00; 95% CI − 0.01 to 0.01, p = 0.6270. These results indicate that the measurement bias was independent of kidney volume, and the agreement between the imaging modalities remained consistent across all volume ranges

US-EL displayed a systematic bias in RKV measurements (p = 0.0211) and TKV measurements (p = 0.0328) but not in LKV measurements (p = 0.4927) (Fig. 3, Table 1).

**Table 1:** Systematic bias in ultrasound ellipsoid (US-EL) measurements of right kidney volume, left kidney volume, and total kidney volume (TKV) compared to the reference standard of magnetic resonance imaging ellipsoid (MRI-EL)

Volume	Mean (95%CI) bias, %	Limit (95%CI), %		P (H_0_: Mean = 0)
		Lower	Upper	
Right	7.5 (1.2 to 13.7)	− 26.6 (− 37.4 to − 15.8)	41.5 (30.7 to 52.3)	0.0211
Left	2.3 (-4.5 to 9.2)	− 35.1 (− 47.0 to − 23.2)	39.8 (27.9 to 51.7)	0.4927
TKV	6.2 (0.5 to 11.8)	− 24.5 (− 34.3 to − 14.8)	36.9 (27.1 to 46.6)	0.0328

Analyses of the within-patient percentage volume difference as a function of volume showed that bias remained relatively consistent across all measured volumes. The mean slopes were not statistically different from zero, indicating no significant variation with volume [RKV: − 0.00; 95% CI − 0.01 to 0.01, p = 0.8874; LKV: 0.01; 95% CI − 0.01 to 0.02, p = 0.4885; Total kidney volume (TKV): − 0.00; 95% CI − 0.01 to 0.01, p = 0.6270]. Bland–Altman analysis revealed that the mean slopes were not significantly different from zero, indicating that there was no substantial variation in the bias with respect to volume (Fig. 3).

**Fig. 4:**
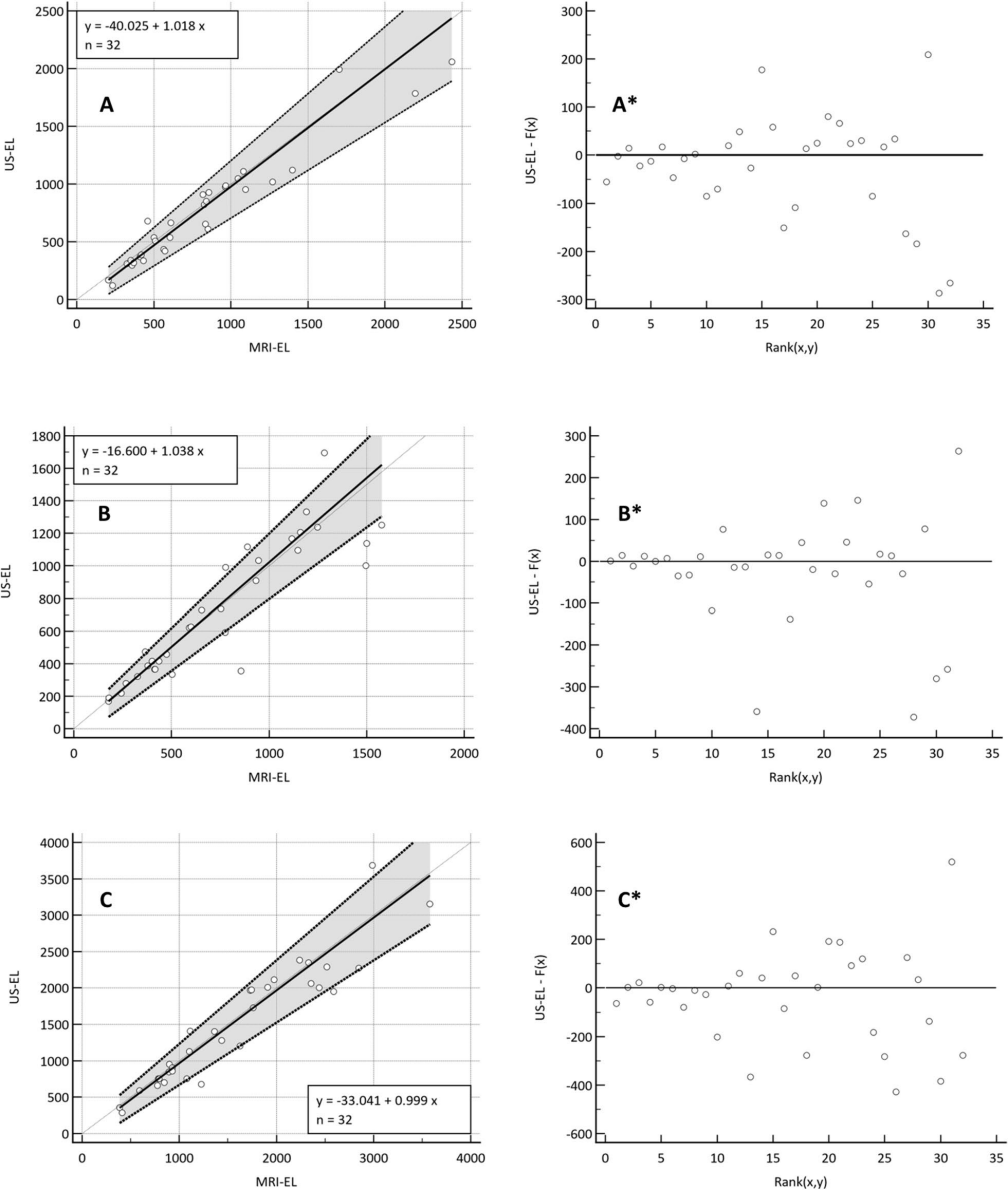
Comparison of right kidney volume (RKV), left kidney volume (LKV), and total kidney volume (TKV) measurements between magnetic resonance imaging using the ellipsoid formula (MRI-EL) and ultrasound with the ellipsoid formula (US-EL), analyzed using the Passing–Bablok regression method. The results are presented as scatter plots with corresponding regression lines and equations. In these equations, the intercept represents the constant measurement error, while the slope reflects the proportional measurement error. A. Right Kidney Volume (RKV): The regression line equation is y = − 40.0 + 1.0xy = − 40.0 + 1.0x, with a 95% confidence interval (CI) for the intercept of − 123.6 to 42.1 and for the slope of 0.83 to 1.16, indicating strong agreement between methods. The accompanying residual plot (A*) illustrates the distribution of differences around the fitted regression line. B. Left Kidney Volume (LKV): The regression line equation is y = − 16.6 + 1.0xy = − 16.6 + 1.0x, with a 95% CI for the intercept of − 84.6 to 35.8 and for the slope of 0.88 to 1.16, demonstrating good agreement. The residual plot (B*) highlights the distribution of differences relative to the fitted regression line. C. Total Kidney Volume (TKV): The regression line equation is y = − 33.0 + 1.0xy = − 33.0 + 1.0x, with a 95% CI for the intercept of − 184.9 to 84.0 and for the slope of 0.85 to 1.15, confirming good agreement. The residual plot (C*) depicts the differences distributed around the regression line

The results comparing RKV, LKV, and TKV between MRI-EL and US-EL, using Passing and Bablok regression analysis, are shown in Fig. 4 and Table 2.

**Table 2:** Passing–Bablok regression analysis between magnetic resonance imaging ellipsoid (MRI-EL) and ultrasound ellipsoid (US-EL) in the right, left, and total kidney volume

	Systematic differences	Proportional differences	Random differences	Linear model validity	Correlation**	
	Intercept (95%CI)^a^	Slope (95%CI)^a^	RSD (± 1.96 RSD interval)	Cusum test for linearity*	CC (95%CI)	p
RKV	− 40.0 (− 123.6 to 42.1)	1.02 (0.83 to 1.16)	111.2 (− 217.9 to 217.9)	0.38	0.96 (0.92 to 0.98)	< 0.0001
LKV	− 16.6 (− 84.6 to 35.8)	1.04 (0.88 to 1.16)	139.1 (− 272.7 to 272.7)	1.00	0.91 (0.82 to 0.96)	< 0.0001
TKV	− 33.0 (− 184.9 to 84.0)	1.00 (0.85 to 1.15)	204.9 (− 401.6 to 401.6)	0.67	0.94 (0.87 to 0.97)	< 0.0001

*CI* confidence interval, *RSD* residual standard deviation, *RKV* right kidney volume, *LKV* left kidney volume, *TKV* total kidney volume

*p > 0.05 means that there is linear relationship between the two measurements and therefore the Passing–Bablok method is applicable

**Spearman rank correlation coefficient

^a^Bootstrap confidence interval (1000 iterations; random number seed: 978)

The Spearman correlation coefficient was 0.96 (95%CI 0.92 to 0.98); 0.91 (95%CI 0.82 to 0.96), and 0.94 (95%CI 0.87 to 0.97) in the RKV, LKV, and TKV, respectively; p < 0.0001 each, respectively (Fig. 4 and Table 2).

These results suggested that the measurement bias did not exhibit a dependency on kidney volume, and the agreement between the imaging modalities was consistent across the entire range of volumes.

CCC of RKV, LKV, and TKV measurements were 0.95, 0.89, and 0.94, respectively (Table 3).

**Table 3:** Concordance correlation coefficient (CCC) between magnetic resonance imaging ellipsoid (MRI-EL) and ultrasound ellipsoid (US-EL) in the right, left, and total kidney volume

	MRI-EL (Reference)		
	Overall study sample (n = 32)		
	CCC (95%CI)	Precision*	Accuracy**
US-EL RKV	0.95 (0.91 to 0.98)	0.96	0.99
US-EL LKV	0.89 (0.80 to 0.95)	0.90	1.00
US-EL TKV	0.94 (0.88 to 0.97)	0.94	0.99

*CCC* concordance correlation coefficient, *CI* confidence interval, *MRI-EL* magnetic resonance imaging ellipsoid, *US-EL* ultrasound ellipsoid, *RKV* right kidney volume, *LKV* left kidney volume, *TKV* total kidney volume

*Pearson correlation coefficient

**It is a bias correction factor that measures how far the best-fit line deviates from the 45° line through the origin (i.e. a value of 1.00 means perfect concordance)

**Fig. 5:**
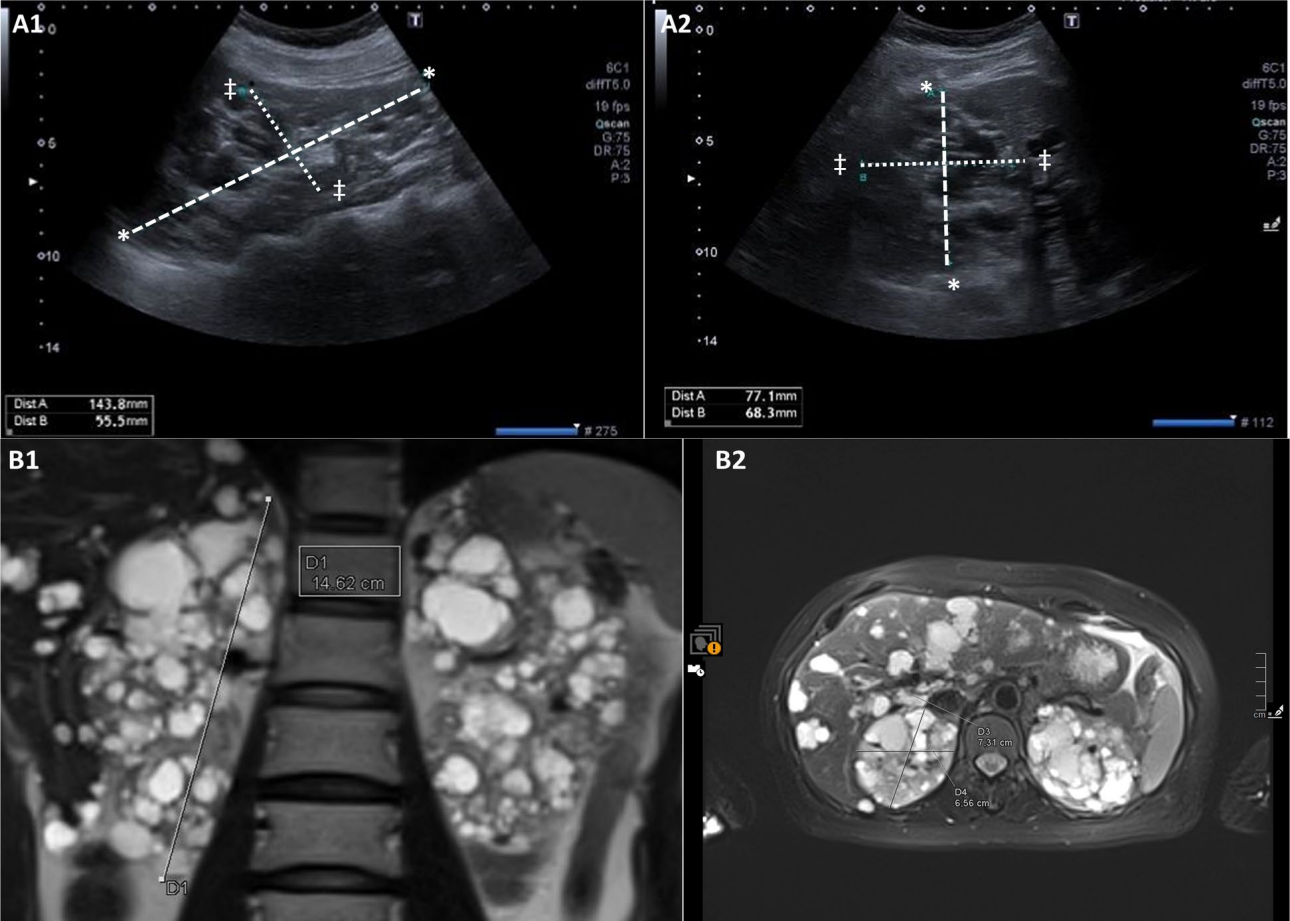
Kidney volume measurement of a 54-year-old female patient with autosomal-dominant polycystic kidney disease (ADPKD). **A** Kidney Volume Measurement Using Ultrasound. 1. Right Kidney Cranio-Caudal and Anterior–Posterior Diameters (Ellipsoid Formula). Cranio-Caudal Distance (marked * to *): 143.8 mm. Anterior–Posterior Distance (marked ‡ to ‡): 55.5 mm. 2. Right Kidney Antero-Posterior and Transverse Diameters (Ellipsoid Formula). Antero-Posterior Distance: 78.1 mm. Transverse Distance: 68.3 mm. **B** Kidney Volume Measurement Using Magnetic Resonance Imaging (MRI). 1. MRI T2 Coronal Cranio-Caudal Diameters (Ellipsoid Formula). Cranio-Caudal Distance D1: 146.2 mm. 2. MRI T2 Axial Anterior–Posterior and Transverse diameter for ellipsoid formula. Anterior–Posterior Distance D3: 73.1 mm. Transverse Distance D4: 65.6 mm

**Fig. 6:**
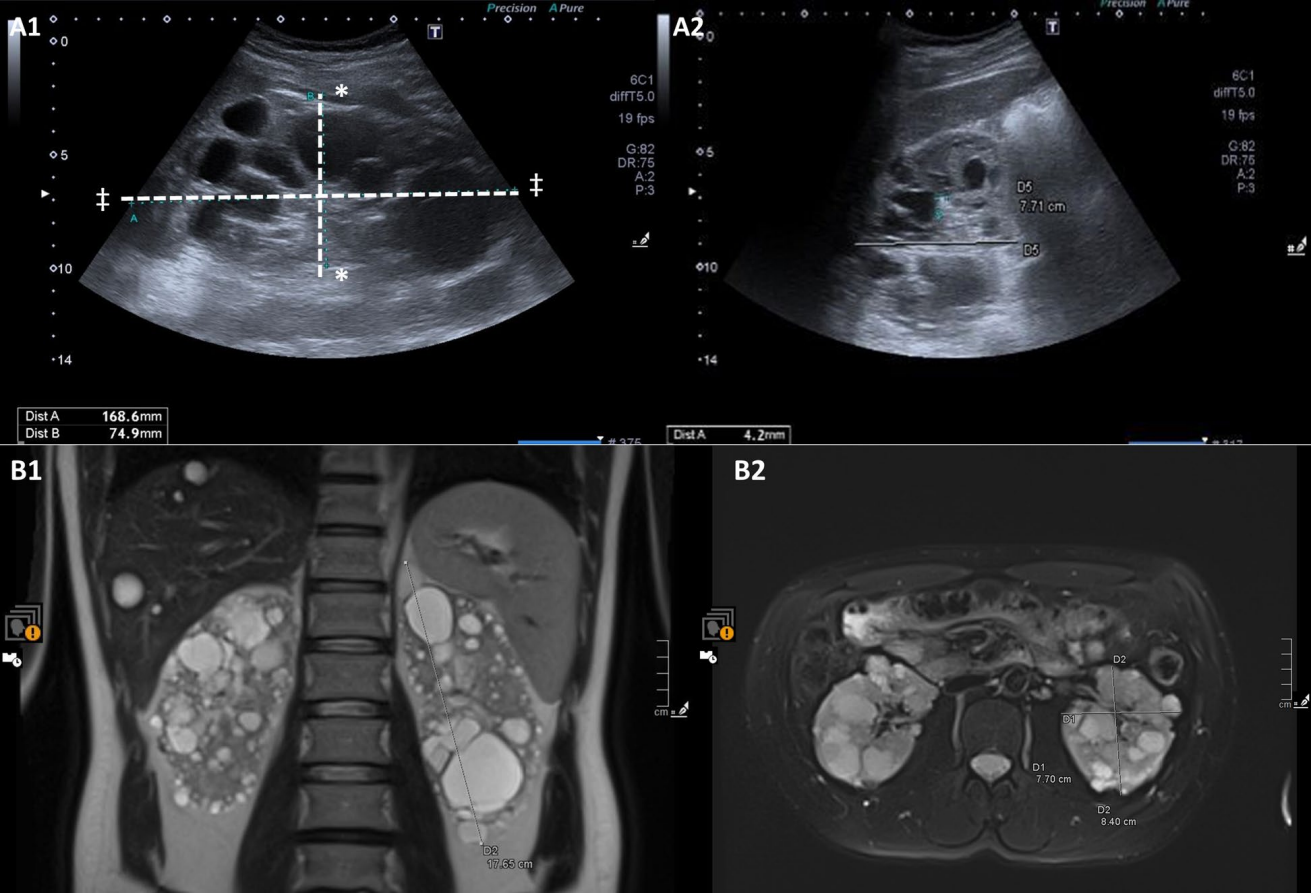
Kidney volume measurement of a 39-year-old male patient with autosomal-dominant polycystic kidney disease (ADPKD). **A** Kidney Volume Measurement Using Ultrasound. 1. Right Kidney Cranio-Caudal and Anterior–Posterior Diameters (Ellipsoid Formula). Cranio-Caudal Distance (marked * to *): 74.9.mm. Anterior–Posterior Distance (marked ‡ to ‡): 168.6 mm. 2. Right Kidney Antero-Posterior and Transverse Diameters (Ellipsoid Formula). Transverse Distance (D5): 77.1 mm. **B** Kidney Volume Measurement Using Magnetic Resonance Imaging (MRI). 1. MRI T2 Coronal Cranio-Caudal Diameters (Ellipsoid Formula). Cranio-Caudal Distance D1: 176.5 mm. 2. MRI T2 Axial Anterior–Posterior and Transverse diameter for ellipsoid formula. Anterior–Posterior Distance D2: 84.0 mm. Transverse Distance D1: 77.0 mm

Figures 5 and 6 illustrate cases of patients with ADPKD, comparing the US-EL measurements with those obtained from MRI-EL.

### Relationship between measured glomerular filtration rate and total kidney volume

A total of 26 subjects had mGFR data available at the time of MRI-EL and US-EL.

The mGFR showed a statistically significant negative correlation with TKV measured by both MRI-EL (slope: − 0.014; 95%CI − 0.027 to − 0.001; p = 0.0281) and US-EL (slope: − 0.015; 95%CI − 0.028 to − 0.001; p = 0.0332) (Figure S1).

### Relationship between estimated glomerular filtration rate and total kidney volume

A total of 26 patients had eGFR data available at the time of MRI-EL and US-EL examinations.

The eGFR demonstrated a statistically significant inverse correlation with TKV as measured by both MRI-EL (slope: − 0.017; 95% CI − 0.029 to − 0.006; p = 0.0054) and US-EL (slope: − 0.019; 95% CI − 0.031 to − 0.007; p = 0.0040) (Figure S2).

## Discussion

The results of the current study found a strong correlation between MRI-EL and US-EL. Blant–Altmant analysis showed low biases in all the measurements. These biases were more pronounced in RKV (7.5%, p = 0.0211) and TKV measurements (6.2%, p = 0.0328), while were very low in the LKV measurements (2.3%, p = 0.4927). It should be noted that if the p-value is less than 0.05, it indicates the presence of a consistent bias, but this does not automatically imply that the methods are not comparable. As noted by Bland and Altman [28], a consistent bias can be easily corrected, if needed, by subtracting the mean difference from the measurements obtained by the US-EL method. Furthermore, it is important to highlight that these differences are independent of renal volume, as the mean slopes did not significantly deviate from zero.

In addition, Passing–Bablok regression analysis comparing MRI-EL and US-EL found strong correlation in RKV (95% CI for intercept − 123.6 to 42.1 and for slope 0.83 to 1.16; ρ = 0.96), LKV (95% CI for intercept − 84.6 to 35.8 and for slope 0.88 to 1.16; ρ = 0.91), and TKV (95% CI for intercept − 184.9 to 84.0 and for slope 0.85 to 1.15; ρ = 0.94). These results clearly indicated that both methods were interchangeable.

Finally, kidney volume concordance between MRI-EL and US-EL, assessed by CCC, found a good agreement in RKV (0.95) and TKV (0.94), but slightly lower concordance in LKV (0.89).

One possible explanation is the different anatomical relationships of the two kidneys. For example, the anatomical proximity of the liver to the right kidney often results in acoustic shadowing during ultrasound examinations. Furthermore, the presence of polycystic liver disease, which is the most common extrarenal manifestation of ADPKD, might influence both imaging and measurements accuracy [29]. In addition, healthy liver parenchyma shows homogeneous echo texture and similar echogenicity compared to the right kidney, which might potentially impact on imaging and measurements [30]. In contrast, the left kidney has fewer surrounding structures that cause such interference, allowing for clearer imaging and more accurate volume measurements [31].

In ADPKD, the cystic burden is most accurately represented by TKV measurements obtained via MRI. Additionally, TKV is currently the most robust predictor of future renal insufficiency in ADPKD [7, 9, 11].

Kidney volume has been evaluated in numerous experimental and clinical studies employing various imaging techniques. MRI provides consistently reproducible measurements of kidney volume, as well as low inter- and intra-operator variability [32], while ultrasound is frequently used due to its accessibility and non-invasive nature [14–16].

While CT and MRI provide superior resolution for detecting small cysts, US remains the preferred initial method due to its accessibility, lower cost, and absence of radiation or contrast exposure. US demonstrates good reproducibility for TKV measurements, correlating well with CT, despite slightly lower accuracy and sensitivity [33]. Additionally, Advances in three-dimensional (3D) US technology have further enhanced diagnostic precision, enabling improved cyst detection and accurate volume measurements [34, 35]. Additionally, artificial intelligence (AI)-assisted 3D US systems show performance comparable to MRI, offering a promising alternative for routine clinical use [35]. These developments underscore the potential of US, particularly 3D and AI-enhanced systems, as accessible and effective tools for monitoring TKV and assessing treatment efficacy in ADPKD [33–36].

Despite being more cost-effective and readily accessible, ultrasound-derived kidney volume measurements are generally considered to be less accurate than those obtained from MRI ellipsoid analysis [34, 37]. Indeed, previous studies have found current US methods are still vulnerable to underestimation compared with MRI- and CT-based estimates [33, 34, 38, 39]. In agreement with these findings, compared with MRI-EL, US-EL displayed systematic bias for underestimating RKV, LKV, and TKV (mean bias of − 7.5%, − 2.3%, and − 6.2%, respectively). Nevertheless, the results of our study (Passing–Bablok regression analysis) showed that the measurement of renal volumes with US-EL was interchangeable with MRI-EL. Therefore, the clinical significance of this underestimation may not be relevant. The increase in kidney size enables clinicians to identify patients experiencing rapid disease worsening, thus supporting timely intervention aimed at slowing disease progression. However, to the best of our knowledge, there is currently no data available in the literature regarding the recommended frequency for performing MRI scans.

Consistent with this hypothesis, Breysem et al. [39] proposed that while US-EL measurements tend to underestimate kidney volume, they still offer a valuable alternative to MRI for the assessment of early ADPKD.

In addition, Bhutani et al. [40] observed that TKV measurements obtained by ultrasound and MRI were comparable, particularly in kidneys of normal to moderate size (< 17 cm). This is likely attributable to the ability to capture the entire kidney within a single imaging plane. Moreover, this study also found that a single measurement of kidney length, either with US or MRI, can reliably predict the development of CKD stage 3 within an 8-year timeframe. This approach effectively reduces healthcare costs while delivering essential prognostic insights into potential outcomes and complications associated with ADPKD [40].

Furthermore, Braconnier et al. [41], reported a strong correlation between ultrasound-measured renal length and MRI-measured renal length in both patients with and without chronic kidney disease (CKD). However, the correlation between MRI and ultrasound measurements for kidney volume, while statistically significant, was notably weaker. Consequently, renal volume assessments should be interpreted with caution [41].

Finally, this study found an inverse correlation between renal function, either assessed by mGFR or eGFR, and TKV, regardless of the method used for determining TKV. Our findings align with those of previous studies, which have demonstrated an inverse correlation between kidney volume and renal function [7, 42–44]. However, these studies were performed evaluating renal volume with MRI, while ours used both MRI and ultrasound, finding no significant differences between both methods. These findings support the use of US-EL for determining kidney volume in clinical practice.

This study has several limitations that should be considered when interpreting its findings. A key limitation of this study is its small sample size of only 32 patients, which restricts the ability to draw generalizable conclusions and limits the broader applicability of the findings. The second major limitation is the time interval between the MRI and ultrasound examinations, which raises the possibility of kidney volume changes occurring during this period. The timing discrepancy between these imaging modalities could influence the findings, as prior research suggests that kidney condition progression is time-sensitive, potentially impacting the consistency of measurements [45]. In our specific case, this delay might be primarily attributed to limited access to MRI facilities. Nevertheless, all patients included in this study had an estimated GFR greater than 60 mL/min (CKD-EPI), indicating early-stage disease, and their clinical stability was maintained throughout the study. Notably, for most patients (18 of 32), the interval between measurements was less than 2 months, with only five patients exceeding 4 months. While renal volume changes cannot be entirely ruled out, no significant clinical alterations were observed that might have influenced the results. Another limitation is that we did not evaluate intraobserver variability of both MRI-EL and US-EL. This study was conducted by a single expert radiologist to ensure consistency and reproducibility. Although US is an operator-dependent technique, and it is advisable that radiologists undergo at least 6 months of specialized training, both techniques have shown low intraobserver variability [39, 41], although such variability may be slightly greater with US-EL [46]. In addition, US may offer other advantages such as low cost, high availability, no radiation exposure, and minimal patient discomfort. Additionally, US is quicker and less expensive than MRI (US takes between 20–30 min and the MRI between 30–50 min) [47].

The primary strength of this study lies in its execution under real-world clinical conditions, providing a more accurate reflection of how these diagnostic tools perform in routine clinical practice, outside of controlled research settings.

## Conclusions

The findings of the current study demonstrated that ultrasound-based ellipsoid kidney volume measurements (including right kidney volume, left kidney volume, and total kidney volume) showed a strong correlation with the corresponding measurements obtained via MRI-based ellipsoid assessment. This suggests that ultrasound, despite its simplicity and greater accessibility, may be considered as a reliable alternative for evaluating kidney volume in daily practice, especially in contexts where MRI may be unavailable or impractical. However, this does not imply that US-EL can be regarded as a complete substitute for MRI-EL.

It would be interesting and probably the subject of future research, to compare the clinical performance of both techniques for monitoring the course of patients with ADPKD. In addition, it might be clinically relevant to analyze the performance of both techniques for predicting the disease’s progression and identifying patients at risk of experiencing accelerated disease progression, which facilitates customized monitoring and tailored treatment strategies.

## Supplementary Information

Supplementary Material 1. Figure S1. Linear regression analysis evaluating the relationship between measured glomerular filtration rate (mGFR) and total kidney volume (TKV) assessed by magnetic resonance imaging ellipsoid (MRI-EL) and ultrasound ellipsoid (US-EL). The shaded grey area represents the 95% confidence interval. The analysis revealed a statistically significant negative correlation between mGFR and TKV for both imaging methods. For MRI-EL, the slope was − 0.014 (95% CI: − 0.027 to − 0.001; p=0.0281), while for US-EL, the slope was − 0.015 (95% CI: − 0.028 to − 0.001; p=0.0332). These findings highlight an inverse relationship between mGFR and TKV, regardless of the imaging modality used.

Supplementary Material 2. Figure S2. Linear regression analysis examining the relationship between estimated glomerular filtration rate (eGFR), assessed by the Chronic Kidney Disease Epidemiology Collaboration (CKD-EPI) formula, and total kidney volume (TKV) assessed by magnetic resonance imaging ellipsoid (MRI-EL) and ultrasound ellipsoid (US-EL). The grey-shaded region represents the 95% confidence interval. The analysis revealed a statistically significant inverse correlation between eGFR and TKV for both imaging methods. For MRI-EL, the slope was − 0.017 (95% CI: − 0.029 to − 0.006; p = 0.0054), while for US-EL, the slope was − 0.019 (95% CI: − 0.031 to − 0.007; p = 0.0040). These results underscore a consistent negative relationship between eGFR and TKV, regardless of the imaging modality employed.

## Data Availability

The data underlying this article will be shared on reasonable request to the corresponding author.

## References

[1] Lanktree MB, Haghighi A, Guiard E, Iliuta IA, Song X, Harris PC, Paterson AD, Pei Y (2018) Prevalence estimates of polycystic kidney and liver disease by population sequencing. J Am Soc Nephrol 29(10):2593–2600 30135240 10.1681/ASN.2018050493 PMC6171271

[2] Ponticelli C, Moroni G, Reggiani F (2023) Autosomal dominant polycystic kidney disease: is there a role for autophagy? Int J Mol Sci 24(19):14666 37834113 10.3390/ijms241914666 PMC10572907

[3] Chebib FT, Torres VE (2016) Autosomal dominant polycystic kidney disease: core curriculum 2016. Am J Kidney Dis 67:792–810 26530876 10.1053/j.ajkd.2015.07.037 PMC4837006

[4] Lanktree MB, Haghighi A, di Bari I, Song X, Pei Y (2021) Insights into autosomal dominant polycystic kidney disease from genetic studies. Clin J Am Soc Nephrol 16(5):790–799 32690722 10.2215/CJN.02320220 PMC8259493

[5] Cornec-Le Gall E, Audrézet MP, Renaudineau E, Hourmant M, Charasse C, Michez E et al (2017) PKD2-related autosomal dominant polycystic kidney disease: prevalence; clinical presentation; mutation spectrum; and prognosis. Am J Kidney Dis 70(4):476–485 28356211 10.1053/j.ajkd.2017.01.046 PMC5610929

[6] Mahboob, M.; Rout, P.; Leslie, S.W.; Bokhari, S.RA. Autosomal Dominant Polycystic Kidney Disease. 2024 Mar 20. In: StatPearls. Treasure Island (FL): StatPearls Publishing; 2024. https://www.ncbi.nlm.nih.gov/books/NBK532934/. Accessed 23 Sept 2024. 30422529

[7] Grantham JJ, Torres VE, Chapman AB, Guay-Woodford LM, Bae KT, King BF, CRISP Investigators et al (2006) Volume progression in polycystic kidney disease. N Engl J Med 354(20):2122–2130 16707749 10.1056/NEJMoa054341

[8] Grantham JJ, Mulamalla S, Swenson-Fields KI (2011) Why kidneys fail in autosomal dominant polycystic kidney disease. Nat Rev Nephrol 7(10):556–566 21862990 10.1038/nrneph.2011.109

[9] Yu ASL, Shen C, Landsittel DP, Harris PC, Torres VE, Mrug M, Consortium for Radiologic Imaging Studies of Polycystic Kidney Disease (CRISP) et al (2018) Baseline total kidney volume and the rate of kidney growth are associated with chronic kidney disease progression in Autosomal Dominant Polycystic Kidney Disease. Kidney Int 93(3):691–699 29290310 10.1016/j.kint.2017.09.027 PMC5826779

[10] Perrone RD, Mouksassi MS, Romero K, Czerwiec FS, Chapman AB, Gitomer BY et al. Total kidney volume is a prognostic biomarker of renal function decline and progression to end-stage renal disease in patients with autosomal dominant polycystic kidney disease. Kidney Int Rep. 2017;2(3):442–450. Erratum in: Kidney Int Rep. 2018;3(4):1015. 10.1016/j.ekir.2017.01.003 PMC5678856 29142971

[11] Irazabal MV, Rangel LJ, Bergstralh EJ, Osborn SL, Harmon AJ, Sundsbak JL, CRISP Investigators et al (2015) Imaging classification of autosomal dominant polycystic kidney disease: a simple model for selecting patients for clinical trials. J Am Soc Nephrol 26(1):160–172 24904092 10.1681/ASN.2013101138 PMC4279733

[12] Shi B, Akbari P, Pourafkari M, Iliuta IA, Guiard E, Quist CF et al (2019) Prognostic performance of kidney volume measurement for polycystic kidney disease: a comparative study of ellipsoid vs. manual segmentation. Sci Rep 9(1):10996 31358787 10.1038/s41598-019-47206-4 PMC6662759

[13] Alam A, Dahl NK, Lipschutz JH, Rossetti S, Smith P, Sapir D et al (2015) Total kidney volume in autosomal dominant polycystic kidney disease: a biomarker of disease progression and therapeutic efficacy. Am J Kidney Dis 66(4):564–576 25960302 10.1053/j.ajkd.2015.01.030

[14] O’Neill WC, Robbin ML, Bae KT, Grantham JJ, Chapman AB, Guay-Woodford LM et al (2005) Sonographic assessment of the severity and progression of autosomal dominant polycystic kidney disease: the Consortium of Renal Imaging Studies in Polycystic Kidney Disease (CRISP). Am J Kidney Dis 46(6):1058–1064 16310571 10.1053/j.ajkd.2005.08.026

[15] Brancaforte A, Serantoni S, Silva Barbosa F, Di Leo G, Sardanelli F, Cornalba GP (2011) Renal volume assessment with 3D ultrasound. Radiol Med 116(7):1095–1104 21643638 10.1007/s11547-011-0691-8

[16] de Amorim Paiva CC, de Mello Junior CF, Guimarães Filho HA, de Brito Gomes CA, Junior LR, Junior GM et al (2014) Reproducibility of renal volume measurement in adults using 3-dimensional sonography. J Ultrasound Med 33(3):431–435 24567454 10.7863/ultra.33.3.431

[17] Pei Y, Obaji J, Dupuis A, Paterson AD, Magistroni R, Dicks E et al (2009) Unified criteria for ultrasonographic diagnosis of ADPKD. J Am Soc Nephrol 20(1):205–212 18945943 10.1681/ASN.2008050507 PMC2615723

[18] Pei Y, Hwang YH, Conklin J, Sundsbak JL, Heyer CM, Chan W et al (2015) Imaging-based diagnosis of autosomal dominant polycystic kidney disease. J Am Soc Nephrol 26(3):746–753 25074509 10.1681/ASN.2014030297 PMC4341484

[19] Grantham JJ (2008) Clinical practice. Autosomal dominant polycystic kidney disease. N Engl J Med 359(14):1477–1485 18832246 10.1056/NEJMcp0804458

[20] Luis-Lima S, Gaspari F, Negrín-Mena N, Carrara F, Díaz-Martín L, Jiménez-Sosa A et al (2018) Iohexol plasma clearance simplified by dried blood spot testing. Nephrol Dial Transplant 33:1597–1603 29211858 10.1093/ndt/gfx323

[21] Krutzén E, Bäck SE, Nilsson-Ehle I, Nilsson-Ehle P (1984) Plasma clearance of a new contrast agent; iohexol: a method for the assessment of glomerular filtration rate. J Lab Clin Med 104:955–961 6438261

[22] Levey AS, Stevens LA, Schmid CH, Zhang YL, Castro AF 3rd, Feldman HI et al. CKD-EPI (Chronic Kidney Disease Epidemiology Collaboration). A new equation to estimate glomerular filtration rate. Ann Intern Med. 2009;150:604–612. Erratum in: Ann Intern Med. 20;155:408. 10.7326/0003-4819-150-9-200905050-00006 PMC2763564 19414839

[23] Higashihara E, Nutahara K, Okegawa T, Tanbo M, Hara H, Miyazaki I et al (2015) Kidney volume estimations with ellipsoid equations by magnetic resonance imaging in autosomal dominant polycystic kidney disease. Nephron 129(4):253–262 25895545 10.1159/000381476

[24] Passing H, Bablok W (1983) A new biometrical procedure for testing the equality of measurements from two different analytical methods. Application of linear regression procedures for method comparison studies in clinical chemistry; Part I. J Clin Chem Clin Biochem 21(11):709–720 6655447 10.1515/cclm.1983.21.11.709

[25] Passing H, Bablok W (1984) Comparison of several regression procedures for method comparison studies and determination of sample sizes. Application of linear regression procedures for method comparison studies in Clinical Chemistry; Part II. J Clin Chem Clin Biochem 22(6):431–445 6481307 10.1515/cclm.1984.22.6.431

[26] Lin LI (1989) A concordance correlation coefficient to evaluate reproducibility. Biometrics 45(1):255–268 2720055

[27] McBride GB. A proposal for strength-of-agreement criteria for Lin’s Concordance Correlation Coefficient. NIWA Client Report: HAM2005-062. Published in 2005. https://www.medcalc.org/download/pdf/McBride2005.pdf. Accessed 2 Sept 2024.

[28] Bland JM, Altman DG (1999) Measuring agreement in method comparison studies. Stat Methods Med Res 8(2):135–160 10501650 10.1177/096228029900800204

[29] Duijzer R, Boerrigter MM, Gevers TJG, Drenth JPH (2024) The pathophysiology of polycystic liver disease. J Hepatol 80(6):981–983 38599980 10.1016/j.jhep.2023.12.027

[30] Di Martino M, Koryukova K, Bezzi M, Catalano C (2017) Imaging features of non-alcoholic fatty liver disease in children and adolescents. Children (Basel) 4(8):73 28800087 10.3390/children4080073 PMC5575595

[31] Hansen KL, Nielsen MB, Ewertsen C (2015) Ultrasonography of the kidney: a pictorial review. Diagnostics (Basel) 6(1):2 26838799 10.3390/diagnostics6010002 PMC4808817

[32] Sharma K, Caroli A, Quach LV, Petzold K, Bozzetto M, Serra AL et al (2017) Kidney volume measurement methods for clinical studies on autosomal dominant polycystic kidney disease. PLoS ONE 12(5):e0178488 28558028 10.1371/journal.pone.0178488 PMC5448775

[33] Hammoud S, Tissier AM, Elie C, Pousset M, Knebelman B, Joly D et al (2015) Ultrasonographic renal volume measurements in early autosomal dominant polycystic disease: comparison with CT-scan renal volume calculations. Diagn Interv Imaging 96(1):65–71 25547671 10.1016/j.diii.2013.04.002

[34] Akbari P, Nasri F, Deng SX, Khowaja S, Lee SH, Warnica W et al (2022) Total kidney volume measurements in ADPKD by 3D and ellipsoid ultrasound in comparison with magnetic resonance imaging. Clin J Am Soc Nephrol 17(6):827–834 35383043 10.2215/CJN.14931121 PMC9269662

[35] Jagtap JM, Gregory AV, Homes HL, Wright DE, Edwards ME, Akkus Z et al (2022) Automated measurement of total kidney volume from 3D ultrasound images of patients affected by polycystic kidney disease and comparison to MR measurements. Abdom Radiol (NY) 47:2408–2419 35476147 10.1007/s00261-022-03521-5 PMC9226108

[36] Breysem L, De Keyzer F, Schellekens P, Dachy A, De Rechter S, Janssens P, CRISP Consortium et al (2023) Risk severity model for pediatric autosomal dominant polycystic kidney disease using 3D ultrasound volumetry. Clin J Am Soc Nephrol 18(5):581–591 36800517 10.2215/CJN.0000000000000122 PMC10278786

[37] Magistroni R, Corsi C, Martí T, Torra R (2018) A review of the imaging techniques for measuring kidney and cyst volume in establishing autosomal dominant polycystic kidney disease progression. Am J Nephrol 48(1):67–78 30071518 10.1159/000491022

[38] Turco D, Busutti M, Mignani R, Magistroni R, Corsi C (2017) Comparison of total kidney volume quantification methods in autosomal dominant polycystic disease for a comprehensive disease assessment. Am J Nephrol 45(5):373–379 28315882 10.1159/000466709

[39] Breysem L, De Rechter S, De Keyzer F, Smet MH, Bammens B, Van Dyck M et al (2018) 3DUS as an alternative to MRI for measuring renal volume in children with autosomal dominant polycystic kidney disease. Pediatr Nephrol 33(5):827–835 29306987 10.1007/s00467-017-3862-6

[40] Bhutani H, Smith V, Rahbari-Oskoui F, Mittal A, Grantham JJ, Torres VE et al (2015) A comparison of ultrasound and magnetic resonance imaging shows that kidney length predicts chronic kidney disease in autosomal dominant polycystic kidney disease. Kidney Int 88(1):146–151 25830764 10.1038/ki.2015.71 PMC4490113

[41] Braconnier P, Piskunowicz M, Vakilzadeh N, Müller ME, Zürcher E, Burnier M et al (2020) How reliable is renal ultrasound to measure renal length and volume in patients with chronic kidney disease compared with magnetic resonance imaging? Acta Radiol 61(1):117–127 31091970 10.1177/0284185119847680

[42] Chapman AB, Guay-Woodford LM, Grantham JJ, Torres VE, Bae KT, Baumgarten DA, Consortium for Radiologic Imaging Studies of Polycystic Kidney Disease cohort et al (2003) Renal structure in early autosomal-dominant polycystic kidney disease (ADPKD): the Consortium for Radiologic Imaging Studies of Polycystic Kidney Disease (CRISP) cohort. Kidney Int 64(3):1035–1045 12911554 10.1046/j.1523-1755.2003.00185.x

[43] Jo WR, Kim SH, Kim KW, Suh CH, Kim JK, Kim H et al (2017) Correlations between renal function and the total kidney volume measured on imaging for autosomal dominant polycystic kidney disease: a systematic review and meta-analysis. Eur J Radiol 95:56–65 28987699 10.1016/j.ejrad.2017.07.023

[44] Phakdeekitcharoen B, Treesinchai W, Wibulpolprasert P, Boongird S, Klytrayong P (2021) The correlation between kidney volume and measured glomerular filtration rate in an Asian ADPKD population: a prospective cohort study. BMC Nephrol 22(1):178 33992075 10.1186/s12882-021-02392-0 PMC8126117

[45] Katagiri D, Wang F, Gore JC, Harris RC, Takahashi T (2021) Clinical and experimental approaches for imaging of acute kidney injury. Clin Exp Nephrol 25(7):685–699 33835326 10.1007/s10157-021-02055-2 PMC8154759

[46] Bakker J, Olree M, Kaatee R, de Lange EE, Moons KG, Beutler JJ et al (1999) Renal volume measurements: accuracy and repeatability of US compared with that of MR imaging. Radiology 211(3):623–628 10352583 10.1148/radiology.211.3.r99jn19623

[47] Bierig S, Jones A (2009) Accuracy and cost comparison of ultrasound versus alternative imaging modalities, including CT, MR, PET, and angiography. J Diagn Med Sonogr 25:138–144

