# Supplementary figures and images for: Ultrasound versus magnetic resonance imaging for calculating total kidney volume in patients with ADPKD: a real-world data analysis
Source: Ultrasound J. 2025 Feb 11;17:13. doi: 10.1186/s13089-025-00400-0 (PMC11814422; doi:10.1186/s13089-025-00400-0)

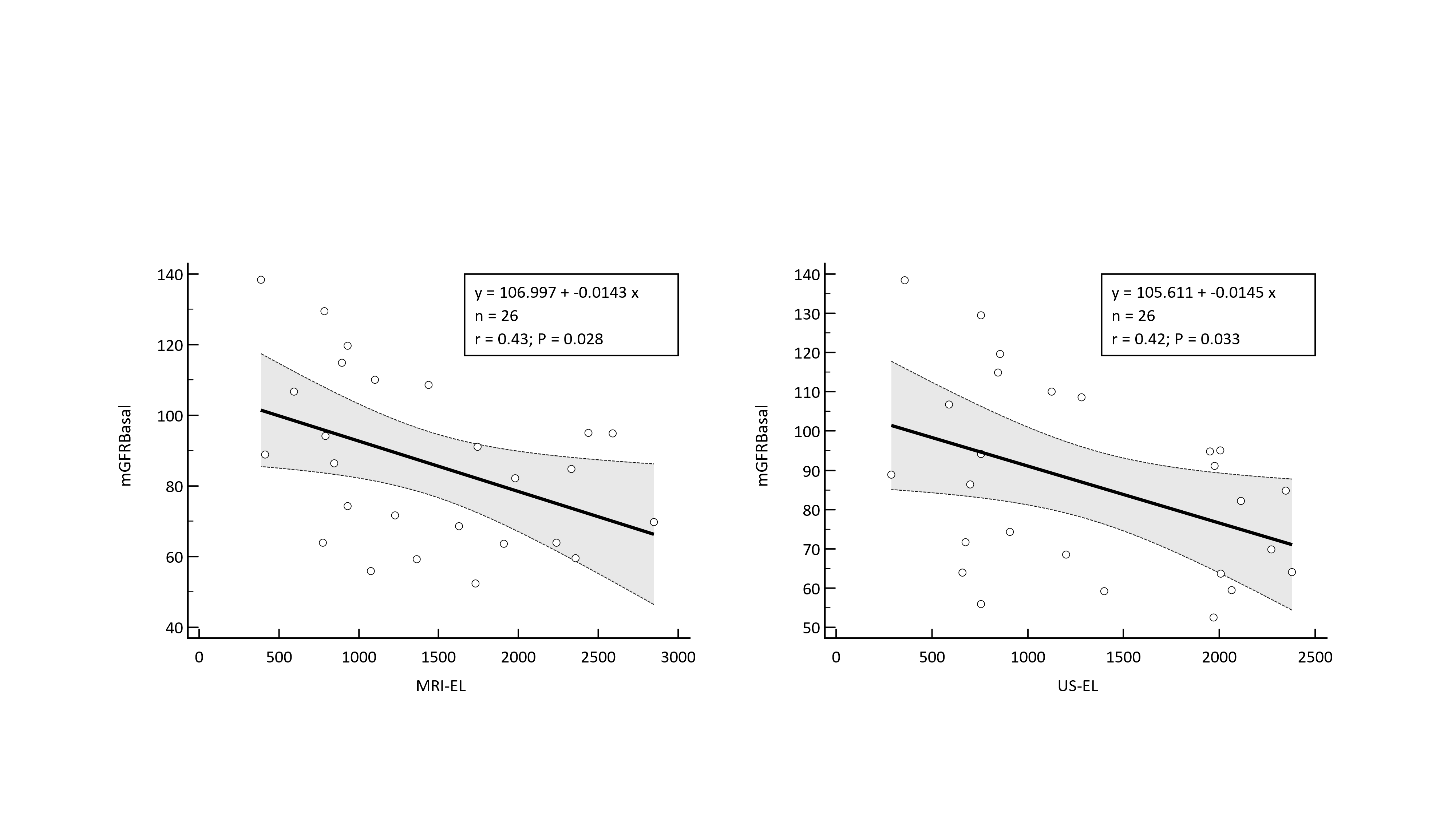

Supplement: Supplementary file 1 — Supplementary Material 1. Figure S1. Linear regression analysis evaluating the relationship between measured glomerular filtration rate (mGFR) and total kidney volume (TKV) assessed by magnetic resonance imaging ellipsoid (MRI-EL) and ultrasound ellipsoid (US-EL). The shaded grey area represents the 95% confidence interval. The analysis revealed a statistically significant negative correlation between mGFR and TKV for both imaging methods. For MRI-EL, the slope was − 0.014 (95% CI: − 0.027 to − 0.001; p=0.0281), while for US-EL, the slope was − 0.015 (95% CI: − 0.028 to − 0.001; p=0.0332). These findings highlight an inverse relationship between mGFR and TKV, regardless of the imaging modality used. [file 13089_2025_400_MOESM1_ESM.tif]

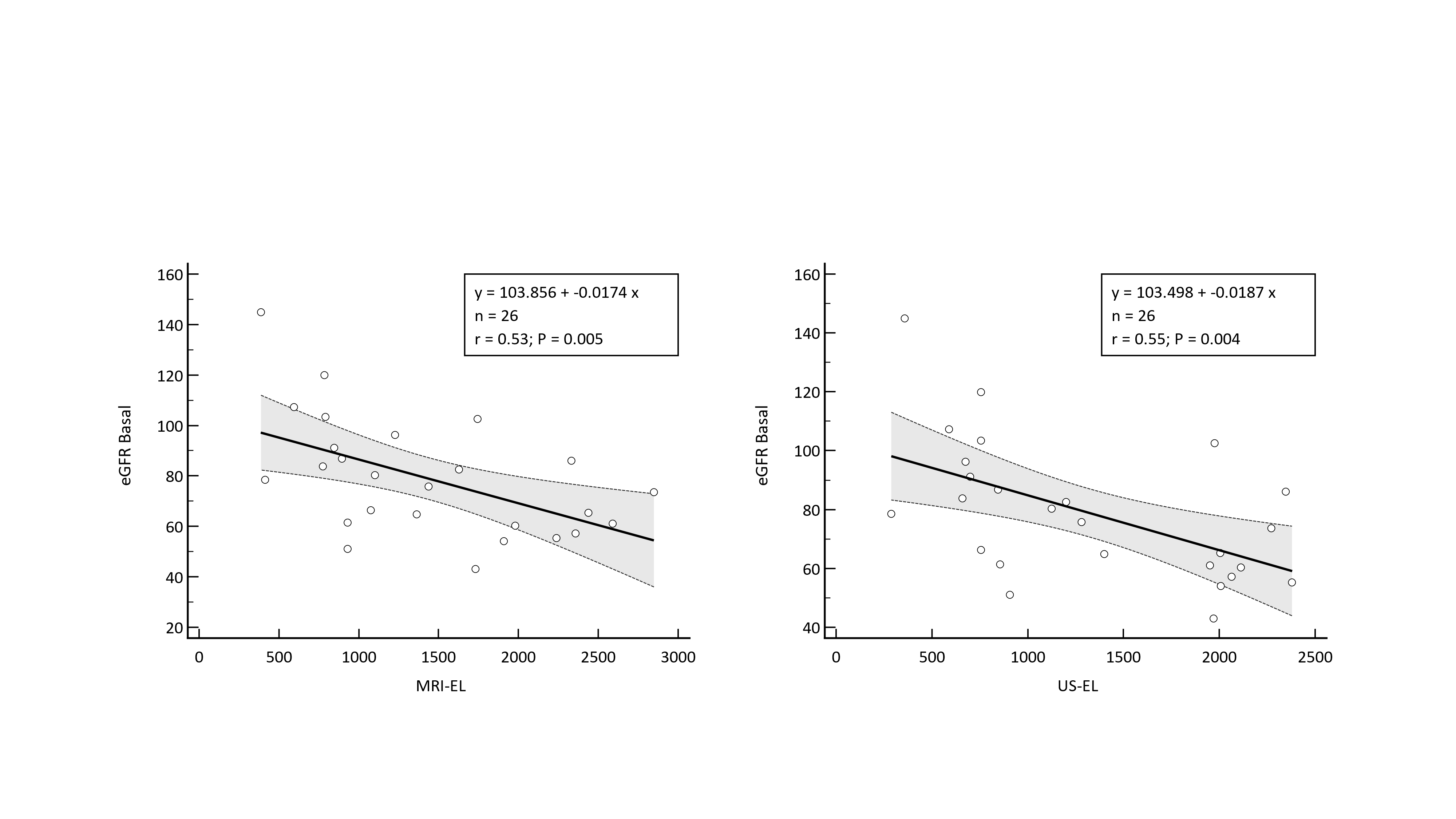

Supplement: Supplementary file 2 — Supplementary Material 2. Figure S2. Linear regression analysis examining the relationship between estimated glomerular filtration rate (eGFR), assessed by the Chronic Kidney Disease Epidemiology Collaboration (CKD-EPI) formula, and total kidney volume (TKV) assessed by magnetic resonance imaging ellipsoid (MRI-EL) and ultrasound ellipsoid (US-EL). The grey-shaded region represents the 95% confidence interval. The analysis revealed a statistically significant inverse correlation between eGFR and TKV for both imaging methods. For MRI-EL, the slope was − 0.017 (95% CI: − 0.029 to − 0.006; p = 0.0054), while for US-EL, the slope was − 0.019 (95% CI: − 0.031 to − 0.007; p = 0.0040). These results underscore a consistent negative relationship between eGFR and TKV, regardless of the imaging modality employed. [file 13089_2025_400_MOESM2_ESM.tif]
